# Effects of Intermittent Administration of Parathyroid Hormone and Parathyroid Hormone‐Related Protein on Fracture Healing: A Narrative Review of Animal and Human Studies

**DOI:** 10.1002/jbm4.10250

**Published:** 2019-11-22

**Authors:** Junro Yamashita, Laurie K McCauley

**Affiliations:** ^1^ Center for Regenerative Medicine, Fukuoka Dental College Fukuoka Japan; ^2^ Department of Periodontics and Oral Medicine, School of Dentistry University of Michigan Ann Arbor MI USA; ^3^ Department of Pathology, Medical School University of Michigan Ann Arbor MI USA

**Keywords:** ANABOLICS, FRACTURE HEALING, ORTHOPEDICS

## Abstract

Intermittent administration of parathyroid hormone (PTH) stimulates skeletal remodeling and is a potent anabolic agent in bone. PTH‐related protein (PTHrP) is anabolic acting on the same PTH1 receptor and is in therapeutic use for osteoporosis. The body of literature for PTH actions in fracture healing is emerging with promising yet not entirely consistent results. The objective of this review was to perform a literature analysis to extract up‐to‐date knowledge on the effects of intermittent PTH and PTHrP therapy in bone fracture healing. A literature search of the PubMed database was performed. Clinical case studies and articles related to “regeneration,” “implant,” and “distraction osteogenesis” were excluded. A narrative review was performed to deliberate the therapeutic potential of intermittent PTH administration on fracture healing. A smaller number of studies centered on the use of PTHrP or a PTHrP analog were also reviewed. Animal studies clearly show that intermittent PTH therapy promotes fracture healing and revealed the strong therapeutic potential of PTH. Human subject studies were fewer and not as consistent as the animal studies yet provide insight into the potential of intermittent PTH administration on fracture healing. Differences in outcomes for animal and human studies appear to be attributed partly to variable doses, fracture sites, age, remodeling patterns, and bone architectures, although other factors are involved. Future studies to examine the dose, timing, and duration of PTH administration will be necessary to further delineate the therapeutic potential of PTH for fracture healing in humans. © 2019 The Authors. *JBMR Plus* published by Wiley Periodicals, Inc. on behalf of American Society for Bone and Mineral Research.

## Introduction

The fracture healing process begins with hematoma formation followed by an inflammatory phase, a proliferative phase with soft callus formation, callus ossification, and finally the bone remodeling phase. The periosteal and endosteal osteoprogenitors along with neovascularization play a crucial role in the early phase of repair. Hence, pathological conditions that have negative influences on progenitor cell recruitment, angiogenesis, or extracellular matrix formation could result in impaired or delayed healing. Such conditions include but are not limited to radiation therapy, osteoporosis, diabetes mellitus (DM), antiangiogenic therapy, advanced age, steroid therapy, and infection.^1^ In long bones, nearly 10% of fractures are associated with impaired healing.^2^, ^3^ Bone grafting procedures or local applications of osteoinductive cytokines, such as bone morphogenetic protein‐2 and ‐7, are applied to promote repair of fractures with compromised healing.^4^ However, these procedures generally involve surgical intervention and therefore have a risk of potential infection and morbidity. For this reason, the establishment of noninvasive therapy to promote healing in situations of compromised fracture repair is of great importance. Systemic anabolic agents including parathyroid hormone (PTH) and PTH‐related protein (PTHrP) have demonstrated potential as candidates that could augment localized osseous healing.

PTH plays a crucial role in calcium and phosphorus homeostasis and has anabolic effects in bone when administered intermittently.^5^ Daily low‐dose injections stimulate osteoblastic bone formation together with osteoclast activation. Neer and colleagues investigated the effects of daily PTH injections on bone fractures in postmenopausal women with prior vertebral fractures and found that daily injections significantly increased vertebral, femoral, and total‐body bone mineral density, and decreased the risk of bone fractures compared with placebo.^6^ Likewise, PTHrP, which binds to the same receptor (PTH1R) as PTH, exhibits a bone anabolic effect when administered intermittently.^7^ Because of their anabolic actions, teriparatide (recombinant human PTH consisting of the first 34 amino acids from the N‐terminus) and abaloparatide (34 amino acid synthetic PTHrP analog) have been approved by the US Food and Drug Administration (FDA) for the treatment of osteoporosis.^8^ Daily teriparatide injections for 24 months in postmenopausal women with severe osteoporosis significantly decrease the incidence of new vertebral fractures compared with antiresorptive therapy with risedronate for 24 months.^9^ Daily abaloparatide injections for 18 months in postmenopausal women with severe osteoporosis significantly reduced the risk of new vertebral and nonvertebral fractures compared with placebo.^10^ Miller and colleagues further reported that the fracture‐protective effect of abaloparatide was comparable to that of teriparatide. Because PTH and PTHrP both bind to PTH1R and activate downstream signaling pathways to stimulate the recruitment and proliferation of osteoprogenitors, they have potential as noninvasive systemic therapies to promote fracture healing. This review highlights the effects of intermittent PTH and PTHrP administration in bone fracture healing.

## Methods

A PubMed search was performed using terms including “parathyroid hormone,” “fracture healing,” “bone,” and “intermittent administration.” Articles related to “regeneration,” “fusion,” “implant,” and “distraction osteogenesis” and clinical case studies were excluded. Although the consideration of PTH or PTHrP in such other instances has merit, they are not discussed in this review in order to focus on fracture healing. In addition, they are covered elsewhere.^11^, ^12^, ^13^, ^14^, ^15^ Abstracts and the full text of the matched articles were collected. References cited in the identified articles were also screened for inclusion. All of the identified articles were reviewed to consider the therapeutic potential of intermittent PTH or PTHrP administration on fracture healing.

## Results

### Animal studies

#### *Nonpathological fractures*

The effects of intermittent PTH administration on physiological fracture healing have been well studied in animals particularly focusing on the appendicular skeleton (Table 1).

**Table 1:** Animal Studies—PTH and Nonpathological Fracture Healing

Animal	Study design	Findings	Reference
W Rats (3 mo) ♀, *n* = 120	Closed unilateral tibial shaft fractures; PTH (1‐34) (60 μg/kg/d) vs. PTH (1‐34) (200 μg/kg/d) vs. VC; duration: 20 or 40 days	Higher PTH dose significantly increased callus volume and strength at d20 and d40. Lower PTH dose did not influence healing at d20 but significantly increased callus volume and strength at d40.	Andreassen et al.,^16^ 1999
SD Rats (3 mo) ♂, *n* = 20	Closed unilateral femoral shaft fractures; PTH (1‐34) (80 μg/kg/d) vs. VC; duration: 21 days	PTH significantly increased callus area, new bone formation, and strength. DXA and pQCT showed that PTH increased density at the fracture site.	Holzer et al.,^17^ 1999
SD Rats (2 mo) ♂, *n* = 136	Closed unilateral femoral shaft fractures; PTH (1‐34) (10 μg/kg/d) vs. VC; duration: 2, 4, 7, 14, 21, 28, and 42 days	PTH significantly increased BMC, BMD, and strength of calluses. PTH increased PCNA(+) osteoprogenitors and TRAP(+) cells in the calluses at d7. PTH upregulated expression of *Col1a1*, *Sparc*, *Alpl*, and *Bglap*.	Nakajima et al.,^18^ 2002
SD Rats (450~550 g) ♂, *n* = 24	Bilateral femoral shaft segmental osteotomy; a combination of local PTH (1‐34) gene therapy and systemic PTH (1‐34) (40 μg/kg) therapy; duration: 6 weeks	The combination of systemic and local PTH (1‐34) treatments enhanced bony healing vs. individual treatment or controls.	Chen et al.^27^ 2003
W Rats (3 mo) ♀, *n* = 136	Closed unilateral tibial shaft fractures; VC, PTH (1‐34), PTH (1‐31), monocyclic [Leu^27^]‐cyclo[Glu^22^‐Lys^26^PTH (1‐31); all at 60 μg/kg/d; duration: 8 weeks; healing was studied at 8 and 16 weeks post‐op.	PTH substantially increased fracture strength, callus volume, and DXA‐BMC at w8. PTH (1‐31) was less effective than other peptides. Callus DXA‐BMC and strength continued to increase after PTH withdrawal.	Andreassen et al.^21^ 2004
SD Rats (2 mo) ♂, *n* = 88	Closed unilateral femoral shaft fractures; PTH (1‐34) (10 μg/kg/d) vs. VC; duration:28 days; healing was assessed at d2, d4, d7, d14, d28 post‐op.	PTH significantly increased cartilaginous callus size and upregulated the expression of PCNA and *Sox9* in chondroprogenitors at d4 and d7.	Nakazawa et al.,^19^ 2005
SD Rats (~450 g) ♂, *n* = 270	Closed unilateral femoral shaft fractures; PTH (1‐34) (5 μg/kg/d) vs. PTH (1‐34) (30 μg/kg/d) vs. VC; duration: up to 35 days; rats were euthanized at d21, d35, or d84 post‐op.	Lower PTH dose significantly increased strength, BMC, BMD, and callus volume at d35 but a higher dose was effective from d21. Higher PTH dose sustained strength and BMD after PTH withdrawal.	Alkhiary et al.,^22^ 2005
SD Rats (5 wo) ♀, *n* = 105	Bilateral femoral shaft osteotomy; VC, PTH (1‐34) (10 or 30 μg/kg) only before osteotomy, PTH (1‐34) (10 or 30 μg/kg) before and after osteotomy; PTH (1‐34) was given 3 times a week; rats were euthanized at 3, 6, and 12 weeks post‐op.	PTH pretreatment for 3 weeks before osteotomy did not affect healing. PTH treatment (10 and 30 μg/kg) accelerated healing both before and after osteotomy. PTH enhanced the remodeling of woven bone into lamellar bone in calluses.	Komatsubara et al.,^23^ 2005
Cynomolgus monkeys (18–19 yo) ♀, *n* = 17	Unilateral femoral shaft osteotomy; PTH (1‐34) (0.75 μg/kg) vs. PTH (1‐34) (7.5 μg/kg) vs. VC; PTH administration: twice a week; duration: 3 weeks before and 26 weeks after osteotomy	Higher PTH dose significantly increased the mechanical properties of the shaft and mineralization of calluses. PTH decreased callus size and accelerated callus mineralization.	Manabe et al.,^25^ 2007
W Rats (200~250 g) ♀, *n* = not specified	Closed unilateral tibial shaft fractures; PTH (28‐48) (0.2 μg or 0.4 μg), PTH (1‐34) (1.0 μg), or VC was given locally on d4, d5, and d6 post‐op; IL‐6 and IL‐6sR were given on d7, d9, and d11 post‐op.	PTH fragments followed by IL‐6 and IL‐6sR significantly enlarged callus volume. PTH (1‐34) followed by IL‐6 and IL‐6sR increased strength by 300%. PTH (28‐48) followed by IL‐6 and IL‐6sR increased strength by 200%.	Rozen et al.,^130^ 2007
C57BL/6J Mice (10 wo) ♂, *n* = 80	Unilateral tibial shaft osteotomy; loading vs. PTH (1‐34) (30 μg/kg/d) vs. loading + PTH (1‐34) (30 μg/kg/d) vs. VC; cyclic loading was given 5 times a week; duration: 18 days	PTH increased callus mineralization on micro‐CT. Addition of cyclic loading further accelerated healing. The combined therapy significantly increased the BMD and bone volume fraction of the calluses.	Gardner et al.,^131^ 2007
C57BL/6 Mice (8 wo) ♂, *n* = 80	Closed unilateral femoral shaft fractures; PTH (1‐34) (30 μg/kg/d) vs. VC; duration: 14 days; mice were euthanized at d2, d3, d5, d7, d10, d14, d21, and d28.	PTH increased the callus size and the expression of ECM‐associated genes in chondrogenesis. PTH induced the expression of Wnts 4, 5a, 5b, and 10b and promoted nuclear localization of β‐catenin.	Kakar et al.,^20^ 2007
SD Rats (3 mo) ♂, *n* = 24	Closed bilateral femoral shaft fractures; PTH (1‐34) (10 μg/kg/d) with and without ultrasound stimuli (LIPUS) vs. VC with and without LIPUS; duration: 35 days	PTH increased callus BMC without influencing its size, whereas LIPUS increased callus size without influencing its BMC. PTH increased callus maturity and strength, whereas LIPUS decreased callus maturity.	Warden et al.,^132^ 2009
W Rats (3 mo) ♂, *n* = 108	Closed femoral shaft fractures or open femoral osteotomy; PTH (1‐34) (50 μg/kg, 5 days a week) vs. VC; duration: 6 weeks	In closed fractures, union rate was 100% in PTH and VC groups. In open fractures, union rate was significantly lower in both groups. PTH failed to increase the union rate in open fractures.	Tӓgil et al.,^28^ 2010
SD Rats (6 mo) ♀, *n* = 72	Ulnar stress fractures; PTH (1‐34) (40 μg/kg/d) vs. alendronate (2 μg/kg/d) vs. VC; rats were euthanized at 2, 4, and 8 weeks after the fracture.	PTH significantly stimulated bone formation at w4 and enhanced strength at w8. Alendronate significantly suppressed the bone formation rate at w4 vs. VC group.	Sloan et al.,^133^ 2010
NZ white rabbits (3.1~3.5 kg) ♀, *n* = 12	A surgical cartilage defect and microfractures in the knee trochlea; PTH (1‐34) (10 μg/kg/d) for 1 or 4 weeks vs. VC for a week; healing was assessed at 3 months post‐op.	VC group had the best healing on gross and histologic analyses. PTH for either 1 or 4 weeks inhibited cartilage regeneration.	Feeley et al.,^134^ 2010
SD Rats (550~600 g) ♂, *n* = 29	Open unilateral mandibular osteotomy; PTH (1‐34) (10 μg/kg/d) vs. no treatment; duration: 7 or 21 days	PTH significantly increased callus formation and radiographic bone density at d7 but not d21. Histologic assessment showed a trend of better bone formation in the PTH group vs. controls.	Rowshan et al.,^37^ 2010
CD1 mice (4~6 wo) ♀, *n* = 44	Closed unilateral tibial shaft fractures without rigid fixation; TPTD (4, 20, or 40 μg/kg/d or 40 μg/kg every 3rd day) vs. VC; duration: 28 days	TPTD (40 μg/kg/d) accelerated callus mineralization from d9. At d15, the micro‐hardness of calluses became similar to that of intact bone.	Mognetti et al.,^135^ 2011
C57BL/6 mice, *n* = 30 (10 wo), ♀	Closed unilateral femoral shaft fractures; PTH (1‐34) (10, 40, or 200 μg/kg/d) vs. VC; duration: 28 days	PTH dose‐dependently stimulated bone formation. PTH did not increase bone stiffness in a dose‐dependent manner.	Milstrey et al.,^136^ 2017
ICR mice (7 wo) *n* = 40	Unilateral femoral shaft osteotomy; a low vs. high dose of TPTD; a low‐ vs. high‐frequency administration	TPTD at a higher dose and/or higher frequency increased callus volume but not strength. TPTD at a lower dose and/or lower frequency increased strength.	Ota et al.,^137^ 2018
C57BL/6J mice, ♂ (2 mo)	Unilateral tibial shaft osteotomy; continuous TPTD (40 μg/kg/d) vs. VC; duration: 2 weeks	Continuous TPTD significantly increased the callus area vs. VC. Continuous TPTD increased strength at d21 vs. VC.	Yutaka et al.,^93^ 2018

SD Rats = Sprague Dawley rats; W Rats = Wistar rats; VC = vehicle control; DXA = dual‐energy X‐ray absorptiometry; pQCT = peripheral quantitative computed tomography; OVX = ovariectomy; OP = osteoporosis; BMC = bone mineral content; BMD = bone mineral density; PCNA = proliferating cell nuclear antigen; TRAP = tartrate‐resistant acid phosphatase; COL1A1 = type I collagen; ON = osteonectin; ALP = alkaline phosphatase; OC = osteocalcin; IL‐6sR = IL‐6 soluble receptor; ECM = extracellular matrix; TPTD = teriparatide [rhPTH (1‐34)].

Work by Andreassen and colleagues is one of the earliest studies that investigated the effect of PTH (1‐34) daily administration on callus formation and mechanical strength advancement of closed tibial shaft fractures in 3‐month‐old female rats after 20 and 40 days of healing.^16^ Two different PTH doses were tested: 60 μg/kg/d, considered a normal dose for rat bone anabolic experiments, and 200 μg/kg/d was set as a high dose. With the 60 μg/kg/d dose, the callus size and strength after 20 days of healing were not different from those of controls, however, they were increased significantly after 40 days of healing. With the higher 200 μg/kg/d dose, the callus size and strength were significantly increased at days 20 and 40 compared with controls. Although strength increased substantially from day 20 to 40 with the higher dose, the callus size decreased ~30% possibly indicating that callus maturation occurred faster with the higher dose. Although the higher dose resulted in a rapid increase in strength, no differences were noted in strength at day 40 between the different doses. Beneficial effects of daily PTH therapy on fracture healing was also reported by Holzer and colleagues where they induced closed femoral shaft fractures in 3‐month‐old female rats and administered 80 μg/kg PTH daily for 21 days.^17^ Significantly higher callus area and strength were found in the PTH‐treated fractures compared with controls. The histopathological assessment revealed a noticeable difference where central ossification of the callus was noted in PTH‐treated fractures, whereas a fibrous union in the callus was found mostly in controls. These two pioneering studies clearly indicate that intermittent PTH administration promotes fracture healing in rats.

#### *Dose effects and mechanisms*

Since the doses used in the above studies were relatively high, the question remained whether a lower PTH dose, more relevant to a human dose, could have a similar effect on fracture healing. In 2002, Nakajima and colleagues performed a study to address this question.^18^ Two‐month‐old male rats received closed femoral shaft fractures and daily 10 μg/kg PTH (1‐34) was administered for up to 42 days. Seven days post‐fracture, the callus was formed radiographically in the PTH group but not in controls. At day 14, the calluses were visible in both groups with enhanced formation in the PTH group. At days 28 and 42, the control calluses exhibited radiolucencies, whereas in the PTH group, radiopacity of the calluses continued to increase, suggesting that PTH stimulated callus formation earlier and enhanced ossification compared with controls. Consistently, the average bone mineral content (BMC), bone mineral density (BMD), and strength of the calluses in the PTH group were significantly higher than controls at days 28 and 42. However, in the contralateral intact femurs, no differences were noted between groups. This suggested that effects of PTH were markedly magnified at fracture sites where bone metabolism and healing were active. The study further investigated the underlying mechanism of the beneficial effect of PTH on fracture healing. Only at day 2 (not days 4, 7, or 14) were significantly increased numbers of proliferating osteoprogenitor cells in the callus noted in the PTH group compared with controls. Collagen type I, alkaline phosphatase, osteocalcin, and osteonectin were significantly upregulated at the RNA level in the callus of the PTH group compared with controls. These results suggest that the mechanisms of PTH benefits on fracture healing are the advancement of callus formation and maturation by the enhanced proliferation of mesenchymal progenitors and their subsequent synthesis of bone matrix proteins. This same group further examined the underlying mechanism of enhanced fracture healing by daily PTH administration.^19^ Closed femoral shaft fractures were made in rats and 10 μg/kg PTH (1‐34) was administered daily for up to 28 days. At days 7, 21, and 28, the cartilage area of the callus was similar between the PTH group and controls, but at day 14 significantly increased cartilage area was noted in the PTH group. Concurrently, PTH treatment significantly upregulated *Sox9* in the callus at day 4 and stimulated proliferation of mesenchymal progenitors significantly at days 4 and 7 in the callus. These results suggest that PTH stimulates chondrogenesis to facilitate endochondral bone repair. Work by Kakar and colleagues confirmed these findings using a mouse fracture healing model.^20^ It was found that PTH treatment induced the expression of chondrogenesis‐related genes (*Sox9, Sox5, Col2a1*, and *Col10*) during early healing (days 5 and 7) and the expression of osteogenesis‐related genes (*Runx2* and *Sp7*) at days 10 and 14. It was further revealed that the levels of nuclear‐localized nonphosphorylated β‐catenin increased in osteogenic and chondrogenic cells in the callus of the PTH group, suggesting that the activation of the Wnt signaling pathway is at least one mechanism of the promoted fracture healing by PTH.

#### *Timing and duration of PTH administration*

PTH accelerates endochondral ossification by stimulating callus formation and remodeling.^19^ It appears early PTH actions may be critical to promote fracture healing; however, the optimal timing and duration of PTH administration was unknown. Andreassen and colleagues evaluated the effect of daily PTH administration (60 μg/kg; 8 weeks) on the healing of closed tibial shaft fractures in 3‐month‐old female rats.^21^ Healing was assessed at weeks 8 and 16 post‐fracture. At week 8, PTH increased fracture strength and callus volume, while callus mechanical quality was similar between groups. At week 16 (treatment daily for 8 weeks followed by 8 weeks without treatment), no differences were noted in fracture strength, callus volume, and callus mechanical quality between groups. From 8 to 16 weeks of healing, fracture strength and callus mechanical quality continued to increase in both controls and PTH withdrawal animals.

Alkhiary and colleagues investigated the effect of PTH withdrawal on subsequent fracture healing.^22^ Closed femoral shaft fractures were created in adult male rats. PTH (1‐34) was administered daily at 5 or 30 μg/kg for 5 weeks, and the study was allowed to continue 7 more weeks. At week 5, both PTH doses (5 and 30 μg/kg/d) significantly increased strength, BMC, and cartilage volume of the fracture site. Cartilage volume, mechanical strength, BMC, and BMD of the fracture site were maintained in the 30 μg/kg PTH group versus controls for 7 weeks after the PTH withdrawal. To determine whether PTH pretreatment had an effect on fracture healing, Komatsubara and colleagues studied young female rats who received intermittent administration of PTH (1‐34) for 3 weeks before femoral shaft transverse osteotomy was performed.^23^ PTH treatment continued up to 12 weeks after osteotomy in half of the PTH‐pretreated animals. In this particular study, PTH was administered 3 times a week at 10 or 30 μg/kg. Both doses of PTH treatment increased lamellar bone formation in the callus 3 weeks after osteotomy. However, this increase was not observed in the PTH‐pretreatment only group or control group. At 12 weeks, strength and cortical shell area were significantly higher in the continuing 30 μg/kg PTH treatment group compared with the PTH‐pretreatment only and control groups. Thus, PTH treatment with a higher dose of 30 μg/kg before and after osteotomy promoted fracture healing. PTH pretreatment itself had no effect on fracture healing. Collectively, the findings from these studies suggest that PTH therapy instituted immediately after a fracture is optimal to enhance healing.

#### *Non‐human primates*

Nearly all animal studies that show the beneficial effects of PTH were performed using rodent fracture healing models. Since bone remodeling in young rodents utilizes different mechanisms than in humans,^24^ it remained unknown whether intermittent PTH therapy would promote fracture healing in animals, which employ Haversian remodeling system as humans do. Work by Manabe and colleagues investigated female cynomolgus monkeys aged 18 to 19 years who received PTH (1‐34) treatment for 29 weeks.^25^ Doses of PTH used were 0.75 μg/kg and 7.5 μg/kg twice a week. PTH administration was initiated 3 weeks before a femoral shaft osteotomy and continued until death at 26 weeks post‐osteotomy. Complete bone union was radiographically observed in the PTH groups as well as controls at 26 weeks after osteotomy. The strength of the fracture was significantly greater in the higher PTH‐dose group than controls. The degree of callus mineralization was significantly greater in the higher PTH‐dose group than the lower PTH‐dose group and controls, indicating that the higher dose of PTH enhanced bone maturation of the callus. Indeed, the callus size was significantly smaller in the higher PTH‐dose group compared with the other two groups. Thus, intermittent PTH therapy, 7.5 μg/kg twice a week, promoted physiological fracture healing in monkeys, which have Haversian remodeling systems.

#### *Long‐bone open fractures*

High‐energy trauma causes open fractures and often results in fracture nonunion,^26^ which is a devastating condition with functional disability, prolonged pain, and financial burden. As of 2003, no study had been published to determine the effect of PTH therapy on open fracture healing and nonunion. Chen and colleagues studied the effect of a combination of local and systemic PTH treatment on the healing responses of fracture nonunion using a rat femoral critical‐sized osteotomy model.^27^ Gene‐activated collagen matrices (GAM) releasing PTH (1‐34) were used for the local PTH treatment, whereas daily subcutaneous injections of PTH (1‐34), 40 μg/kg for 6 weeks were performed as systemic treatment. In this study, systemic PTH therapy promoted bone healing of critical‐sized segmental defects compared with controls. Notably, the anabolic effect of systemic PTH therapy during osteotomy healing was enhanced when combined with local therapy. Tägil and colleagues investigated whether PTH therapy could promote healing of open fractures as it had been shown to do in closed fractures.^28^ Three‐month‐old male rats received either closed femoral shaft fractures or open femoral osteotomy with periosteal stripping. PTH (1‐34), 50 μg/kg or saline was administered 5 days/week for 6 weeks. In closed fractures, union was observed in all animals regardless of treatment, yet PTH treatment significantly increased callus size and strength compared with controls. In open fractures, union was observed in ~2/3 of the animals regardless of treatment. These data demonstrated lower union rates in open versus closed fractures as expected but suggested that PTH therapy is not efficient in promoting healing of open fractures.

#### *Fracture healing in animal models of osteoporosis*

Impaired fracture healing occurs in patients with metabolically compromised conditions, such as osteoporosis, diabetes, and advanced age. Intermittent PTH therapy has positive effects on fracture healing in animals with those conditions (Table 2).

**Table 2:** Animal Studies—PTH and Pathological Fracture Healing

Animal	Study design	Findings	Reference
♀ SD Rats (4 mo) *n* = 75 OP model (OVX)	Closed bilateral tibial shaft fractures; VC, PTH (1‐84) (15 μg/kg/d, 30 days), PTH (1‐84) (150 μg/kg/d, 30 days), 17‐β estradiol (30 μg/kg/d, 30 days)	PTH significantly increased callus size, trabecular bone area, and strength in a dose‐dependent manner. 17‐β estradiol did not influence healing in ovariectomized rats.	Kim and Jahng,^29^ 1999
♀, W Rats (27 mo) *n* = 26, Aging	Closed unilateral tibial shaft fractures; PTH (1‐34) (200 μg/kg/d, 21 or 56 days) vs. VC (21 or 56 days)	PTH treatment significantly increased callus volume, strength, and BMC by pQCT at d21 and d56.	Andreassen et al.,^33^ 2001
♀ W Rats (7 mo) *n* = 40 OP model (OVX)	Cancellous bone osteotomy in the proximal right tibias; PTH (1‐34) (100 μg/kg, once a week) vs. VC in OVX‐ and Sham‐rats; duration: 4 weeks	PTH significantly increased cancellous bone volume and suppressed adipocyte volume. PTH significantly increased PCNA(+) cells in the osteotomy site in both OVX‐ and Sham‐rats.	Nozaka et al.^30^ 2008
♀ SD Rats (3 mo) *n* = 75 OP model (OVX)	Bilateral transverse osteotomy in the tibial metaphysis in OVX and untreated healthy rats; PTH (1‐34) (40 μg/kg/d) administration; d1‐d35 vs. d7‐d35 vs. d14‐d35 vs. d14‐d28	PTH (d1‐d35) and PTH (d7‐d35) improved bone parameters in all rats. Serum OC levels were elevated in PTH‐treated rats. PTH (d14‐d35) and PTH (d14‐d28) were less effective in bone healing.	Komrakova et al.,^138^ 2010
♂ SD Rats (8 mo) *n* = 60 Androgen deficiency	Bilateral transverse surgical osteotomy in the tibial metaphysis; rats with orchiectomy (Orx) and Sham surgery; VC vs. PTH (1‐34) (40 μg/kg/d) vs. PTH (1‐34) (40 μg/kg, every other day)	PTH had no adverse effect on muscle metabolism enzymes. PTH increased callus area and densities similarly in Sham‐ and Orx‐ rats. PTH administered every other day was less effective in healing.	Komrakova et al.,^139^ 2011
♀ SD Rats (3 mo) OVX *n* = 145	Closed Unilateral tibial shaft fractures; PTH (1‐34) (60 μg/kg, 3 times a week) vs. ZA (1.5 μg/kg/w) vs. PTH (1‐34) + ZA vs. VC; duration: 4 or 8 weeks	ZA + PTH significantly enhanced fracture healing vs. monotherapy. In contralateral tibiae, PTH, ZA, and ZA + PTH were not significantly different.	Li et al.,^140^ 2012
♀ SD Rats (25 wo) n > 60 OVX	Right legs: paralysis by botulinum toxin‐A; closed right tibial shaft fracture; PTH (1‐34) (20 μg/kg/d) vs. VC; duration: 8 weeks	Muscle paralysis significantly reduced callus area, BMD, and BMC. PTH had beneficial effects on callus volume and strength regardless of paralysis of the legs.	Ellegaard et al.,^141^ 2013
♀ SD Rats (7 mo) OVX *n* = 105	Unilateral tibial metaphyseal osteotomy; PTH (1‐34) (30 μg/kg, 3 times a week) vs. VC; duration: 3 or 5 weeks post‐op	PTH significantly increased bone volume and promoted union. PTH significantly increased Runx2(+) cells but not PCNA(+) or Sox9(+) cells.	Tsuchie et al.,^31^ 2013
♀ SD Rats (3 mo) *n* = 32 OVX	Bilateral surgical tibial shaft fractures; PTH (1‐34) (30 μg/kg, 3 days a week) vs. ultrasound stimuli (LIPUS) vs. PTH + LIPUS vs. VC; duration: 6 weeks	PTH and PTH + LIPUS showed significantly higher BMD and trabecular bone integrity. Mechanical properties were significantly higher in LIPUS, PTH and PTH + LIPUS groups.	Mansjur et al.,^142^ 2016
♀ SD Rats (2 mo) *n* = 60 OVX, T2DM	Closed unilateral femoral shaft fractures; VC, OVX, and OVX + T2DM rats; effects of PTH (1‐34) (50 μg/kg, 5 days a week) and insulin were evaluated.	Bone volume fraction, trabecular connectivity density, and the cartilaginous callus area ratio were significantly increased in insulin and PTH + insulin groups.	Liu et al.,^36^ 2017
♀ Athymic rats, *n* = 36 (3 mo)	Surgical multiple rib segmental defects; VC vs. TPTD (4 μg/kg) vs. hMSCs (5 injections) vs. TPTD + hMSCs	PTH + hMSCs significantly increased bone volume at w4. At w8, complete bone bridging was 35% and 6.5% in TPTD + hMSCs and TPTD groups, respectively.	Cohn Yakubovich et al.,^143^ 2017

OVX = ovariectomy; ZA = zoledronic acid; T2DM = type II diabetic mellitus; hMSCs = human mesenchymal stem cells.

Kim and Jahng induced estrogen deficiency in 4‐month‐old female rats by performing ovariectomy.^29^ Three months after ovariectomy, animals demonstrated reduced bone mass. Closed tibial shaft fractures were then created, and PTH (1‐84), 15 μg/kg or 150 μg/kg, 17‐β estradiol (30 μg/kg), or saline was administered daily for 30 days to test if PTH treatment promoted healing of bone fractures in an osteoporotic‐like animal model. Callus trabecular bone ratio and mechanical strength were significantly lower in ovariectomized rats than sham‐operated rats, showing impaired fracture healing in ovariectomized rats. PTH treatment, regardless of the dose, significantly increased trabecular area and callus mechanical strength compared with controls. The trabecular bone area in the callus with the higher PTH dose was similar to that in sham‐operated rats. No effect of 17‐β estradiol on fracture healing was noted in gonadectomized osteopenic animals. The results of the study supported that PTH therapy promoted fracture healing in gonadectomized osteopenic animals. Later, Nozaka and colleagues and Tsuchie and colleagues reported that PTH therapy exerted its beneficial effects on healing not only in long bone shaft fractures but also in cancellous bone osteotomy in gonadectomized osteopenic animals.^30^, ^31^

#### *Advanced age*

Fracture healing is often compromised in patients with advanced age. It has been reported that proliferation and osteoblastogenesis of mesenchymal stem cells decline age‐dependently,^32^ which would contribute to compromised fracture healing in patients with advanced age. Since intermittent PTH administration increases osteoblast progenitors indirectly via increased expression of insulin‐like growth factor 1 and fibroblast growth factor 2 in the bone marrow environment, it was expected that PTH would promote fracture healing in aged animals. Andreassen and colleagues examined the response of fracture healing to PTH therapy in aged animals.^33^ Closed tibial shaft fractures were created in 27‐month‐old female rats. PTH (1‐34) 200 μg/kg was administered daily for 3 or 8 weeks and resulted in significantly increased mechanical strength and callus bone mineral content at both timepoints compared with controls. External callus volume was also significantly increased in the PTH group versus controls. These results clearly support the PTH promotion of fracture healing in aged animals. The results of the study were compared with their previous study published in 1999.^16^ In control rats, callus formation rate was slower in old animals compared with young animals. Callus volumes at 8 weeks after fractures in old animals were similar to that in young animals at 20 days. When animals were treated with PTH, callus volumes at days 20 or 21 were similar between the young and old animals. However, callus volumes declined after 20 days of healing in the young animals, whereas callus volumes remain unchanged from weeks 3 to 8 in the old animals. This assessment suggested that intermittent PTH administration is capable of promoting fracture healing in aged animals but with a less robust response than found in young animals.

#### *Diabetic condition*

Compromised fracture healing is found in patients with diabetes mellitus (DM).^34^ Postmenopausal type 2 DM (T2DM) dysregulates bone metabolism and is associated with cortical porosity and trabecular defects.^35^ The effect of PTH therapy on fracture healing in the diabetic condition was studied using ovariectomized rats with T2DM.^36^ T2DM was induced in female rats by high‐fat diet feeding and a single injection of low‐dose streptozotocin. Ovariectomy was performed after the establishment of T2DM. Six weeks after ovariectomy, closed femoral shaft fractures were made. PTH (1‐34) (50 μg/kg) was administered 5 days a week in addition to insulin and healing was evaluated at 2 and 3 weeks post‐fracture via radiographic, microcomputed tomographic, and histomorphometric assessment. T2DM impaired fracture healing significantly compared with controls at weeks 2 and 3. Insulin therapy significantly promoted fracture healing compared with T2DM controls. The combination therapy of PTH and insulin further enhanced fracture healing. Significantly increased bony callus area, BMD, and bone volume fraction were noted in the combination therapy versus the insulin alone group. Findings of the study indicate that intermittent PTH administration strongly enhanced fracture healing in rats with T2DM.

#### *Maxillofacial bones*

Almost all studies using animals with PTH effects on fracture healing have been performed in long bones, which typically heal via the process of endochondral ossification. Most of the maxillofacial bones develop by intramembranous ossification in which mesenchymal tissue directly converts into bone without intermediate cartilage formation. Because the ossification process is distinct between maxillofacial and long bones, it raises the question of whether PTH administration could exert a beneficial effect in maxillofacial bone fracture healing. Rowshan and colleagues investigated the effect of intermittent PTH administration on mandibular fracture healing in rats.^37^ A mandibular vertical osteotomy was created from the sigmoid notch to the inferior border of the mandible in male rats and was stabilized with an external fixation device. PTH (1‐34) 10 μg/kg was administered daily for 1 and 3 weeks. During healing, rats were fed a soft diet. Densitometric analysis revealed a significant increase in radiopacity in the PTH group at week 1 but not week 3 compared with controls. The histologic assessment showed a general trend toward increased new bone formation in the osteotomy site in the PTH group compared with controls at week 3. The authors concluded that low‐dose intermittent PTH administration might enhance mandibular fracture healing. To our knowledge, this is the only study that investigated the PTH effect on fracture healing in maxillofacial bones. Although the method was sophisticated, the assessment was rather subjective. Hence, conclusions drawn from this study would benefit from further validation.

### Human subject studies

The results of animal studies collectively support that intermittent PTH administration promotes fracture healing. Human case studies report PTH as successful in the treatment of nonunion or delayed union of fracture healing.^38^, ^39^, ^40^, ^41^, ^42^, ^43^ Although the PTH doses used in animal studies were relatively high compared with the doses used in humans, encouraging findings from preclinical studies and case reports prompted clinical trials (Table 3).

**Table 3:** Human Subject Studies—PTH and Fracture Healing

Subject	Study design	Findings	Reference
Postmenopausal women, *n* = 102 Double‐blind RCT	Distal radial fractures with no surgical intervention required; TPTD (20 μg/d) vs. TPTD (40 μg/d) vs. placebo; *n* = 34/group; duration: 8 weeks	TPTD 20 μg/d significantly reduced healing time by 2 weeks vs. placebo. However, TPTD 40 μg/d did not significantly reduce healing time vs. placebo or TPTD 20 μg/d.	Aspenberg et al.,^44^ 2010
Postmenopausal women, *n* = 27 Subgroup analysis of the above study^44^	Distal radial fractures with no surgical intervention required; TPTD (20 μg/d) vs. TPTD (40 μg/d) vs. placebo; callus formation at 5 weeks was radiographically evaluated.	Rich calluses were classified in 9 cases. All cases had been treated with TPTD. A TPTD dose and callus formation were strongly correlated at 5w‐post‐fracture.	Aspenberg and Johansson,^45^ 2010
Osteoporosis, *T*‐score < −2.5 Age > 70 yo, RCT	Unilateral pelvic fracture healing; PTH (1‐84) (100 μg/d, *n* = 21) vs. controls (*n* = 44); all patients received 1000 mg of calcium and 800 IU of vitamin D.	PTH (1‐84) significantly shortened healing time vs. controls (7.8 vs. 12.6 weeks). Healing rate at w8 was 100% for PTH (1‐84) and 9.1% for controls.	Peichl et al.,^46^ 2011
Age > 60 yo, *n* = 19, RCT	Low‐energy hip fractures requiring surgery; alendronate (70 mg/w, *n* = 8) vs. TPTD (20 μg/d, *n* = 6) vs. controls (*n* = 5); duration: 4 weeks	At 6 months, no patients had fracture nonunion in TPTD and alendronate groups, whereas 2 patients developed nonunion in controls.	Kanakaris et al.,^144^ 2015
Age > 50 yo, *n* = 159, RCT	Internally fixed femoral neck fracture healing; TPTD (20 μg/d for 6 months, *n* = 78) vs. placebo (*n* = 81); healing at 24 months was evaluated.	No differences were found in radiographic healing or need for revision surgery in TPTD vs. placebo group at 12 months post‐op.	Bhandari et al.,^48^ 2016
Premenopausal women, *n* = 13 Double‐blind RCT	Lower‐extremity stress fractures; TPTD (20 μg/d) vs. placebo; effects of TPTD on bone biomarkers and healing were assessed; duration: 8 weeks	TPTD administration had significant anabolic effects. TPTD promoted fracture healing vs. placebo but not significantly.	Almirol et al.,^50^ 2016
Postmenopausal women, *n* = 40, RCT	Proximal humerus fractures; TPTD (20 μg/d) vs. no treatment; duration: 4 weeks; radiographic assessment at w7 and w12.	Radiographic assessment in TPTD vs. controls was not significant. No differences were noted for pain and function.	Johansson,^51^ 2016
*T*‐score ≤ −2.0, *n* = 224, RCT	Recent pertrochanteric hip fractures; TPTD (20 μg/d) vs. oral risedronate (35 mg/w); duration: 78 weeks	TPTD resulted in significantly less pain at w18, and significantly shorter time to complete TUG tests at w6, 12, and 18 vs. risedronate.	Malouf‐Sierra et al.,^49^ 2017

RCT = randomized clinical trial; TPTD = teriparatide [rhPTH (1‐34)]; QOL = quality of life; BP = bisphosphonate; TUG test = timed up‐and‐go test.

#### *Distal radius fractures*

Aspenberg and colleagues conducted a multicenter randomized controlled clinical trial to test the hypothesis that daily teriparatide [rhPTH (1‐34)] at the 20 or 40 μg dose would accelerate fracture healing in humans.^44^ Postmenopausal women with a distal radial fracture were recruited. Patients received either teriparatide 20 μg, 40 μg, or placebo daily for 8 weeks and fracture repair was assessed radiographically. The time to complete cortical bridging was on average 7.4, 8.8, and 9.1 weeks for teriparatide 20 and 40 μg and placebo, respectively, with no differences found between groups. The authors then blindly reevaluated the baseline radiographs and excluded 9 patients citing volar dislocation, a previous fracture, and minimal dislocation as reasons for exclusion. A post hoc assessment then revealed that 20 μg of teriparatide had significantly shorter healing time versus placebo and 40 μg teriparatide groups. The study findings suggested that the 20 μg teriparatide dose could accelerate fracture repair, whereas 40 μg had no effect.

Aspenberg and Johansson, who were co‐authors of this study, extracted a subgroup of patients from the cohort for further assessment.^45^ The subgroup consisted of patients treated at the Linköping study site (*n* = 27). In the subgroup analysis, the authors evaluated the callus formation 5 weeks after fracture. Callus formation was arbitrarily classified as rich, intermediate, or poor using radiographs in a blinded manner. There were 9 patients classified as rich, and they all received teriparatide (6 patients with 40 μg and 3 patients with 20 μg). Another 9 patients were classified as poor of which 7 patients received placebo. The subgroup analysis revealed a strong trend that 40 μg of teriparatide had a more prominent positive effect on radiographic callus formation than 20 μg.

#### *Pelvic fractures*

In 2011 Peichl and colleagues investigated the effect of PTH (1‐84) on pelvic‐fracture healing in 65 postmenopausal severe osteoporotic patients.^46^ The patients who had been treated for osteoporosis within 6 months before the PTH (1‐84) treatment were excluded. Twenty‐one patients received a once‐daily injection of 100 μg of PTH (1‐84), and the remaining 44 patients served as untreated controls. CT scanning was taken at baseline and repeated every fourth week until cortical bridging at the fracture site was confirmed. The average time to healing was 7.8 and 12.6 weeks for the PTH group and controls, respectively. PTH treatment accelerated the time to cortical bridging significantly versus controls. At 8 weeks, all fractures healed in the PTH group, whereas only 4 of 44 control fractures healed (*p* < 0.001). The study further evaluated the functional outcome with the use of a timed up‐and‐go (TUG) test and found significantly improved function in the PTH group versus controls. The results of the study strongly indicated that PTH (1‐84) treatment promoted healing of pelvic fractures in elderly patients with severe osteoporosis.

#### *Hip fractures*

Falls often cause a hip fracture in the elderly population, which then results in a dramatic decline in BMD and a slow recovery.^47^ Bhandari and colleagues performed a multicenter randomized controlled clinical study to determine if teriparatide could enhance hip fracture healing.^48^ Teriparatide at 20 μg (*n* = 78) or placebo (*n* = 81) was administered daily for 6 months. The primary endpoint was the occurrence of revision surgery rate at 1‐year post‐fracture. The study found that the rate of revision surgery was not different between groups (17% in the PTH group and 14% in controls). Moreover, no differences were noted in radiographic healing between groups at 10 weeks, 6 months, or 12 months. This particular clinical study indicated that teriparatide failed to reduce the risk of revision surgery and to improve radiographic signs of fracture healing compared with placebo.

Malouf‐Sierra and colleagues reported the results of a multicenter randomized clinical trial that evaluated the effect of teriparatide and risedronate on femoral neck BMD in patients with pertrochanteric hip fracture.^49^ In the study, 20 μg teriparatide was administered daily (*n* = 86), whereas 35 mg oral risedronate was administered once weekly (*n* = 85) for 78 weeks. At study end, teriparatide treatment resulted in a significant increase in the BMD values of the lumbar spine and femoral neck compared with risedronate treatment. Although the study was not intended to analyze fracture healing, it should be noted that teriparatide treatment resulted in significantly less pain at week 18 and better function (faster TUG test) at weeks 6, 12, and 18.

#### *Lower‐extremity stress fractures*

The short‐term effects of teriparatide administration on fracture healing were studied in premenopausal women with lower‐extremity stress fractures (tibia, metatarsal, femoral neck, fibula, and calcaneus).^50^ In this randomized, double‐blinded study, the levels of bone biomarkers such as procollagen type I N‐terminal propeptide (P1NP), osteocalcin, and C‐terminal telopeptide of type I collagen (CTX) were measured before and after teriparatide administration for 8 weeks. Fracture healing was assessed by magnetic resonance imaging (MRI) before and after teriparatide therapy as well. Teriparatide‐treated women with fractures had a significantly greater anabolic window compared with placebo‐treated women with fractures. With the elevated anabolic window at the systemic level, complete or improved fracture repair was noted in 83.3% of women in the teriparatide‐treated group versus 57.1% of women in the placebo‐treated group. Although the differences were not significant at 8 weeks, teriparatide improved fracture healing compared with placebo.

#### *Humerus fractures*

Johansson performed a study to determine the effect of teriparatide on healing of proximal humerus fractures in 40 postmenopausal women who never received bisphosphonates.^51^ Teriparatide at 20 μg was administered once daily for 4 weeks. Pain and radiographs were assessed at baseline and weeks 7 and 12. Radiographic callus formation was arbitrarily classified as “normal” or “better.” No differences were noted in radiographic callus formation, pain, and function between the teriparatide group and controls. Although this study was underpowered, it suggested that at this dose, teriparatide was not able to improve healing of proximal humerus fractures in postmenopausal women.

### Bisphosphonates and fracture healing

Patients who develop osteoporotic bone fractures are often already receiving bisphosphonate therapy before a fracture occurs. Because bisphosphonates are retained in bone for years after the cessation of drugs and continue suppressing bone remodeling, there is a concern that bisphosphonates may hinder fracture healing.^52^ Indeed, atypical femoral fractures (AFF) and osteonecrosis of the jaw (ONJ), both of which are challenging to treat, are associated with suppressed bone turnover in association with bisphosphonates.^53^, ^54^ However, findings from animal studies indicate that while bisphosphonates may delay callus remodeling, overall healing is not hampered.^55^, ^56^ In fact, such treatment increases the size and mechanical strength of calluses.^57^, ^58^ Interestingly, bisphosphonate therapy has an inhibitory effect on primary fracture healing (no callus formation), which happens when bone fragments are rigidly fixed.^59^, ^60^ This suggests that bisphosphonates may not be useful when very rigid fixation is achieved. However, such situations are unlikely to happen in real life.

#### *Effects of teriparatide versus bisphosphonates on healing*

Although PTH and bisphosphonates are used for the treatment of osteoporosis, their actions are distinct. PTH promotes bone turnover, whereas bisphosphonates suppress it. Aspenberg and colleagues compared the therapeutic effects of teriparatide (20 μg/d) with risedronate (35 mg/week) on recovery after pertrochanteric hip fractures.^61^ The medication was started within 2 weeks after the fixation of a fracture and lasted for 26 weeks. The TUG test, hip pain, and radiographic healing were assessed. It was found that teriparatide treatment resulted in less pain and a shorter time to complete the TUG test than risedronate.

A retrospective study was performed to compare the effects of teriparatide (20 μg/d) with alendronate (35 mg/week) on healing of osteoporotic vertebral compression fractures.^62^ Radiographic healing and the rate of surgical interventions were assessed. The study found that teriparatide produced a shorter time‐to‐union and lower incidence of surgical interventions than risedronate treatment.

#### *PTH effects on healing in patients with previous bisphosphonates*

Many patients with osteoporosis receive bisphosphonate therapy. Since bisphosphonates suppress bone remodeling for years even after their cessation, those patients are expected to respond to PTH therapy differently from those without a history of bisphosphonate treatment. The bone anabolic effect of PTH therapy was shown to be blunted when teriparatide was used after or concomitant with alendronate treatment.^63^, ^64^, ^65^ However, recent studies have shown that teriparatide therapy (20 μg/d for 2 years) increased the BMD and bone formation in patients with osteoporosis, irrespective of their history of bisphosphonate treatment.^66^, ^67^, ^68^ Teriparatide administration increased cortical bone formation and turnover and improved cancellous bone microstructure.^69^, ^70^ Thus, teriparatide administration appears to have positive effects on patients with osteoporosis previously treated with bisphosphonates. A retrospective study was conducted to assess the effect of teriparatide on intertrochanteric fractures in 189 osteoporotic patients who received surgical interventions followed by teriparatide administration at 20 μg/d for 6 months.^71^ In this study, patients who received teriparatide had significantly shorter time‐to‐union and reduced complications compared with controls regardless of previous alendronate treatment. Furthermore, the quality of life (QOL) score was significantly better in the teriparatide group at months 3 and 6 versus controls regardless of prior alendronate treatment (Table 4).

**Table 4:** Human Subject Studies—PTH and Fracture Healing in Patients Previously Treated with BPs

Subject	Study design	Findings	Reference
*n* = 189 Retrospective study	Surgically treated osteoporotic intertrochanteric fractures; TPTD (20 μg/d) for 6 months with or without a history of BP treatment vs. controls	TPTD had significantly shorter time‐to‐union, better QOL, and lower‐frequency complications and mortality rates regardless of previous BP treatment.	Huang et al.,^71^ 2016
14 AFF patients Prospective study	5 patients received TPTD (20 μg/d) for 6 months and 9 patients conservative treatment. HRpQCT scans of the distal radius and tibia at BL and 6 months.	TPTD significantly increased bone remodeling markers and resulted in less dense bone at the distal radius and tibia. TPTD promoted fracture healing compared with conservative therapy.	Chiang et al.,^74^ 2013
45 AFF patients (37 Sx and 8 conventional therapy)	37 surgically treated patients: 16 with and 21 without TPTD. Time to healing and frequency of delayed healing or nonunion were assessed.	TPTD treatment significantly shortened time to healing and lowered the frequency of delayed healing or nonunion vs. non‐TPTD treatment.	Miyakoshi et al.,^75^ 2015
13 AFF patients All women	7 patients received TPTD (20 μg/d) for 12 months immediately after AFF and 6 patients received TPTD for 12 months beginning 6 months after AFF. Radiographic healing at 6 and 12 months was assessed.	Superior healing with immediate TPTD therapy vs. delayed therapy. There were lesser BMD declines at the distal 1/3 radius in the immediate vs. delayed group.	Greenspan et al.,^76^ 2018

TPTD = teriparatide [rhPTH (1‐34)]; BP = bisphosphonate; QOL = quality of life; HRpQCT = high‐resolution peripheral micro‐computed tomography.

#### *PTH effects on healing of atypical femoral fractures*

An AFF is a rare complication associated with long‐term bisphosphonate treatment.^54^ The pathobiology of AFFs is not clear, but since the cessation of bisphosphonates reduces the risk of AFFs,^72^, ^73^ suppressed bone turnover is suspected to be involved. Teriparatide, which stimulates bone turnover, has therefore been studied for its potential to promote healing of AFFs. However, a large‐scale study cannot be easily conducted, as AFFs are rare. Chiang and colleagues compared the healing of AFFs with and without teriparatide therapy.^74^ Five patients received teriparatide, while 9 did not. The study found that teriparatide therapy for 6 months significantly increased serum bone remodeling markers and was associated with decreased pain and improved healing. A retrospective study was performed to determine the effect of teriparatide therapy on healing of AFFs. The medical records of 37 patients who received surgical intervention were reviewed, and healing was compared between patients with and without teriparatide therapy.^75^ Time to healing and the frequency of delayed healing or nonunion were assessed. Teriparatide therapy significantly shortened the healing time and resulted in a reduced incidence of delayed healing or nonunion compared with non‐teriparatide therapy. The pro‐healing effect of teriparatide therapy on AFFs was compared between patients with immediate initiation and a delay of 6 months.^76^ In that study, radiographic bone healing at 6 and 12 months was assessed in 13 women with AFFs. The study found that immediate teriparatide initiation promoted healing of AFFs more than delayed initiation did.

### PTH‐related protein (PTHrP)

PTH and PTHrP both bind to the same receptor (PTH1R) with similar bioactivity in their N‐terminal region. This forms a rational foundation that PTHrP would be osteoanabolic as well as benefit fracture healing and several studies have supported this (Table 5).

**Table 5:** Animal Studies—PTHrP and Fracture Healing

Animal	Study design	Findings	Reference
Corticosteroid‐treated rabbits (3.5 kg) ♂, *n* = 30	Bilateral ulnar osteotomies; PTHrP analog RS‐66271 (0.01 mg/kg/d) vs. saline control; healing at 6 weeks was evaluated radiographically and biomechanically.	RS‐66271 administration resulted in significantly enhanced radiographic healing parameters, higher union rate, and greater biomechanical strength vs. controls.	Bostrom et al.,^77^ 2000
*Pthrp* ^+/−^ mice (8 wo) ♂, *n* = 60	Closed mid‐diaphyseal femur fractures; calluses were evaluated at 1, 2, and 4 weeks for healing. Results were compared between *Pthrp* ^+/−^ and *Pthrp* ^+/+^ mice.	Significantly reduced callus size, BMD, and osteoblast numbers at 2 weeks in *Pthrp* ^+/−^ mice. Healing was impaired in *Pthrp* ^+/−^ mice vs. wild type.	Wang et al.,^78^ 2013
Leptin receptor null (*Lepr* ^−/−^) mice (12 wo), *n* = 30	*Lepr* ^−/−^ mice exhibit diabetic phenotype. Closed mid‐diaphyseal femur fractures; PTHrP (80 μg/kg/d) vs. vehicle; duration: 2 weeks	PTHrP significantly increased callus calcified area, BMD, osteoblast numbers, and osteoclast perimeters in *Lepr* ^−/−^ mice vs. wild type. PTHrP promoted fracture repair in *Lepr* ^−/−^ mice.	Liu et al.,^79^ 2015
*Pthrp* ^+/−^ and *Pthrp* ^+/+^ mice (8 wo) *n* = 60	Closed mid‐diaphyseal femur fractures. The effect of PTHrP administration (80 μg/kg/d) in *Pthrp* ^+/−^ was compared with *Pthrp* ^+/+^ mice.	PTHrP administration increased callus areas, endochondral bone formation, and callus remodeling in both *Pthrp* ^+/−^ and *Pthrp* ^+/+^ mice.	Wang et al.,^80^ 2017
C57BL/6J mice (26 g weight) ♂ *n* = 120	Femoral shaft osteotomy; abaloparatide (60 μg/kg/d) vs. teriparatide (15 μg/kg/d) for 28 days; screw implants in the proximal tibiae; abaloparatide vs. teriparatide at various doses for 10 days	Abaloparatide increased the density of calluses vs. teriparatide but not significantly. Increased pull‐out force by abaloparatide was dose‐dependent.	Bernhardsson and Aspenberg,^84^ 2018
SD Rats (12 wo) ♂, *n* = 96	Closed mid‐diaphyseal femur fractures; abaloparatide or saline at 5 or 20 μg/kg/d for 4 or 6 weeks; healing was evaluated by micro‐CT, histomorphometric measurements, and mechanical tests.	Abaloparatide increased total area and new bone formation in calluses vs. controls. Abaloparatide group had greater BV, BV/TV, BMC, and BMD vs. controls. Abaloparatide increased the mechanical properties of calluses.	Lanske et al.,^85^ 2019

BMD = bone marrow density; BV = bone volume; TV = tissue volume; BMC = bone mineral content.

In 2000, Bostrom and colleagues utilized a rabbit ulnar model with a 1 mm defect and superimposed steroid associated compromised wound healing.^77^ A PTHrP analog, RS‐66271 was administered that resulted in an increased radiographic union and increased callus size versus saline controls. Further important work that suggested PTHrP may be beneficial for fracture healing came with a study that demonstrated that PTHrP haploinsufficiency resulted in compromised fracture healing.^78^ In 2015, Liu and colleagues performed a mid‐diaphyseal fracture healing experiment in wild‐type and leptin receptor mutant mice. PTHrP administration for 2 weeks at 80 μg/kg significantly increased callus size to a greater extent in wild‐type than leptin receptor mutant mice.^79^ More recently, a follow‐up study using PTHrP haploinsufficiency mice found that the administration of PTHrP (80 μg/kg) to 8‐week‐old mice with closed mid‐diaphyseal femur fractures resulted in increased callus size and bone mineral density in both wild‐type and haploinsufficiency mice.^80^ These animal studies support findings that are similar to intermittent PTH and PTHrP both facilitating improved fracture healing and overall outcomes.

Renewed interest in PTHrP has surfaced since abaloparatide was approved by the FDA for the treatment of postmenopausal osteoporosis in 2017. Abaloparatide, a PTHrP 1‐34 analog, is identical to PTHrP in the first 20 amino acids and then has 50% similarity in amino acids 21 to 34. Abaloparatide has selective activation of the PTH1R and has been described as having an advantage over teriparatide in that it does not activate osteoclasts to the same extent.^81^ In human studies for postmenopausal osteoporosis with fracture risk as an outcome, abaloparatide significantly reduced fracture risk and increased trabecular bone scores.^10^, ^82^, ^83^ Very few studies have been performed to evaluate the effect of abaloparatide on fracture healing. Bernhardsson and Aspenberg performed the initial study that evaluated both teriparatide and abaloparatide.^84^ Since it is proposed that abaloparatide is not as osteoclast inductive as teriparatide, it would seem that there may be a difference in their actions during osseous wound healing. A comparison of teriparatide and abaloparatide was performed in mouse tibial drill hole defects and femoral osteotomy defects. Multiple doses of both teriparatide and abaloparatide were utilized in the tibial defects with a dose‐dependent increase in pullout strength of tibial steel screws versus controls for both treatments, reflecting benefits to osseous healing. In mice with femoral defects, one dose of teriparatide (15 μg/kg) and abaloparatide (60 μg/kg) was utilized. Control mice had a 23% increase, teriparatide had a 38% increase, and abaloparatide had a 47% increase in callus BV/TV. The callus BV/TV was significantly increased in both teriparatide and abaloparatide groups versus controls but there was no difference between teriparatide and abaloparatide. Further studies with increased clarity on the osteoblastic and osteoclastic responses of both agents relative to fracture healing may be valuable to discern potential clinical applications. The most recent study of abaloparatide and fracture healing was a rat study that compared two doses (5 and 20 μg/kg/d) in 12‐week‐old rats for 4 or 6 weeks.^85^ Abaloparatide treatment resulted in greater callus area and volume, higher bridging scores, and greater callus maximum load and stiffness versus vehicle controls.

As with PTH, preclinical studies using PTHrP or an analog are promising; however, there is a need in general for more mechanistic studies to better strategize therapeutic options. To date, there are no apparent human studies using abaloparatide for the purpose of fracture healing.

### Primary hyperparathyroidism and bone fracture healing

Primary hyperparathyroidism (PHPT) is characterized by hypercalcemia with elevated or inappropriately normal serum PTH levels. PHPT mainly affects the skeleton and kidney, but their involvement is relatively mild in developed countries. Patients with PHPT have a low BMD in cortical bone‐rich sites, such as the distal one‐third radius and femoral neck, while the BMD is spared at trabecular sites such as the lumber spine.^86^, ^87^ Because of these observations, PHPT is thought to have differential effects on bone: catabolic on the cortical and anabolic on the trabecular bone. However, the histomorphometric assessment of iliac crest biopsies from patients with PHPT showed an increased activation frequency, shallower resorption pits, and thinner walls of the trabeculae than in patients without PHPT, indicating that PHPT greatly altered the trabecular architecture.^88^ Indeed, an epidemiological study revealed that patients with PHPT had an increased risk of vertebral, distal forearm, rib, and pelvic fractures compared with those without PHPT.^89^ Even though the BMD values of the spine according to DXA were not altered, vertebral fractures occurred to a similar degree as distal forearm fractures. This raises the question of whether or not PHPT has an anabolic effect on trabecular bone‐rich sites.

Stein and colleagues studied the cortical and trabecular microstructures at the radius and tibia using high‐resolution peripheral quantitative tomography (HR‐pQCT).^90^ Their study found a thinner cortex and more widely spaced and heterogeneously distributed trabeculae in patients with PHPT than in non‐PHPT controls. Such microstructural abnormalities would account for the overall increased fracture risk in PHPT patients. Thus, PHPT deteriorates not only the cortical but also the trabecular bone.^91^, ^92^

Because bone is a target organ of PTH, continuously elevated serum PTH levels in PHPT patients likely affect bone fracture healing. A study showed that continuous teriparatide infusion to mice resulted in delayed healing of tibial fractures.^93^ Although the literature on fracture healing in PHPT patients is sparse, case reports may provide insight into the effect of PHPT on healing.^94^, ^95^, ^96^, ^97^, ^98^ Lancourt and Hochberg reported 4 fracture cases with delayed healing or nonunion in PHPT patients. After the excision of a parathyroid adenoma, the serum calcium levels were corrected, and healing occurred without further delay.^98^ The pro‐healing effect of the removal of a parathyroid adenoma on compromised fracture healing in PHPT patients can be found in other reports as well.^94^, ^95^, ^96^ In contrast, however, Chalmers and Irvine reported that parathyroidectomy or the correction of serum chemistry did not promote healing of hip fractures in 5 elderly patients (average age 76 years) with hyperparathyroidism and osteoporosis.^97^ It should be mentioned that, in those particular cases, the health status of 3 patients was extremely poor, and 1 showed marked bone loss. Such pathological conditions likely have additional negative influences on healing.

## Discussion

The results of animal studies clearly indicate that intermittent PTH administration promotes fracture healing by accelerating callus formation and ossification. The results of human subject studies are also supportive of PTH promoting fracture healing yet are fewer and not as overwhelming. The doses used in animal studies range from 1 to 200 μg/kg/d with the majority being 10, 30, and 40 μg/kg/d. When these doses are converted to human equivalent doses using the body surface area (BSA) normalization method, 10, 30, and 40 μg/kg for rats are comparable to 97, 292, and 389 μg for a human weighing 60 kg.^99^ Although the conversion is based solely on BSA and therefore may not be entirely accurate, the conversion suggests that the doses used in animal studies are high compared with the approved human dose, which is 20 μg/d for the treatment of osteoporosis. Because the animal studies show that PTH administration promoted fracture healing dose‐dependently, differences in outcomes between animal and human studies could in part be attributed to the high doses used in animal studies. Osteoporotic fractures generally occur during the post‐senescence period in humans. Rats reach sexual maturity, adulthood, and reproductive senescence at approximately 50 days, 7 months, and 1.6 years, respectively.^100^ Therefore, the vast majority of animal studies used young adult metabolically active rats. At this life stage of rats (<7 months old), 10 rat days can be considered to be equivalent to 1 human year.^100^ Taking this temporal ratio into account, PTH therapy in animal studies is less frequent with longer duration than that in humans. Furthermore, although rodents and humans share comparable systemic physiology and show similarities in pathogenesis, rodents show 30~50 times faster genomic responses in inflammatory conditions than humans.^101^ Therefore, further investigations are needed to optimize the PTH dose, frequency, and duration for fracture healing in humans. Among different species, great variations in bone micro‐ and macroarchitectures, mineral density, chemical contents, metabolism, remodeling, and healing mechanisms exist.^102^, ^103^, ^104^, ^105^ For instance, the cortex of rat long bones consists of the primary bone tissue with dispersed Haversian systems near the endosteal surface.^106^ In contrast to humans, intracortical or endosteal remodeling is generally absent in rodents when they are young.^107^, ^108^ Since bone anabolism by intermittent PTH administration is accompanied by enhanced bone remodeling, the general lack of intracortical remodeling in young rodents might require a higher PTH dosage to increase bone mass than would be needed in humans. Another possible explanation for the discrepancy in outcome is the difference in fracture sites between animal and human studies. Closed femoral or tibial shaft fractures were predominantly used in animal studies, whereas distal radial, pelvic, hip, and proximal humerus fractures were used in the human studies. Even though the biological process of fracture healing may be similar among those sites, the local biomechanical and biological environment would differ, which could alter the response to PTH therapy. Moreover, radiographs were exclusively used to assess fracture healing in human studies. Since it would be challenging ethically to perform the immunohistochemical, histomorphometric, microcomputed tomographic, or mechanical assessment of a fracture site in humans for research purposes, analytical tools in human studies were not as sensitive as those in animal studies. This might also affect differences in outcomes. Future studies evaluating the impact of PTH or PTHrP on fracture healing at different skeletal sites may provide new insights to anabolic mechanisms of PTH, PTHrP, and their analogs.

PTH therapy affects the entire body even though it is intended to use for the promotion of fracture healing. Therefore, bone mass in nonfracture sites in the same skeleton is increased. However, as shown in a study by Nakajima and colleagues,^18^ fracture sites in young rats show greater anabolic responses to PTH than the contralateral nonfractured bone sites, indicating that the effects of PTH are augmented at fracture sites where bone metabolism is active. Nonetheless, bone mass is increased to some extent in other nonfractured bone sites depending on the duration of PTH therapy and the age of patients. Daily PTH administration is limited to 2 years or less for the treatment of osteoporosis in humans. Although the duration would be shorter if PTH were applied to promote fracture healing, the consequences of discontinuing PTH (ie, rebound) on the skeleton are a concern, especially when elderly patients with systemic diseases receive PTH therapy. In animal studies, the duration of PTH therapy is typically for 3~6 weeks in fracture healing studies as well as bone anabolism studies. Therefore, studies for bone anabolism by PTH can be considered to obtain insight into the consequence of discontinuing PTH on the new bone formed in response to PTH therapy. Studies have shown that the cessation of PTH therapy induces loss of new bone mass in animals.^109^, ^110^ Accumulating evidence indicates that the discontinuation of PTH therapy results in not a rapid but a gradual decline in the BMD over 2 to 3 years in humans.^111^, ^112^, ^113^ Such a decline in the BMD appears to be site‐dependent. A large decrease has been reported for the lumbar spine, which is a trabecular bone‐rich site, while a relatively slow decline has been observed for the femoral neck, total hip, and distal one‐third of the radius.^114^, ^115^, ^116^ Furthermore, the effects of discontinuing PTH may depend on the sex.^117^ Women with osteoporosis may lose a greater portion of their BMD than men with osteoporosis after the cessation of teriparatide. Although bone loss after the cessation of PTH is inevitable, switching to antiresorptive therapy, particularly bisphosphonates, prevents the decline in the bone mass.^63^, ^112^, ^118^ At present, almost all studies on the effects of PTH cessation have investigated changes in the BMD values of the spine, total hip, femoral neck, or distal radius. No studies have yet described how PTH cessation affects the bone mass and quality of healed bone at fracture sites as well as newly formed bone at nonfracture sites in aged patients with systemic diseases. Further studies are necessary to address the post‐treatment sequelae regarding fracture healing once PTH is discontinued.

Osteoporosis is a major health care concern for the elderly population. Roughly 1 in 2 white women and 1 in 5 men are predicted to have an osteoporosis‐related fracture in their lifetime in the US.^119^ As the population ages,^120^ the risk of fractures in the advanced age group, which is associated with high morbidity and mortality rate, should increase.^121^ Therefore, it is important to establish noninvasive therapy to promote healing of compromised fracture repair in long bones and vertebrae. Patients with osteoporosis are often on bisphosphonate treatment. PTH therapy appears to have positive effects on fracture healing in patients previously on bisphosphonates. This suggests that osteoclasts (as bisphosphonate targets) may not play a major role in the early phase of osseous healing.^60^ Indeed, post‐fracture bisphosphonate treatment had no negative effects on healing, although it slowed callus remodeling.^122^ Since PTH showed effectiveness in AFF healing,^74^, ^75^ it may be possible that PTH therapy reverses the suppressed osteoclast activity by bisphosphonates. When the therapeutic effect on fracture healing was compared between teriparatide and bisphosphonates, better outcomes were observed with teriparatide.^61^, ^62^ This may suggest that although osteoclast suppression is not detrimental, better healing is achieved with osteoclast activation. However, as the evidence is not extensive, more research is needed to have a better understanding of osteoclast suppression during osseous healing.

As for craniofacial bones, the majority of fractures in young and elderly populations occur due to traffic accidents, sports, interpersonal violence, or falling.^123^ Complications, such as nonunion, malunion, fibrous union, and infection, occur in maxillofacial fracture healing, particularly in the mandible.^124^ Such complications often result in malocclusion, facial asymmetry, and osteomyelitis, all of which need to be corrected to improve function and quality of life. If PTH promotes craniofacial fracture healing, it would be a strong adjunct therapy. Currently, however, there is only one animal study that investigated PTH effects on craniofacial fracture healing.^37^ In contrast, there have been numerous case reports with positive results for the administration of teriparatide for osseous healing in osteonecrosis of the jaw.^125^, ^126^, ^127^, ^128^, ^129^ Additional investigations will clarify its therapeutic potential for the treatment of maxillofacial fractures and other osseous conditions.

In summary, intermittent administration of PTH increases bone mass and has been approved as a therapeutic agent for the treatment of osteoporosis. Although promising, its optimal therapeutic potential in bone fracture healing is yet unclear. Accumulating evidence indicates that the intermittent administration of PTH promotes fracture healing in animals. However, the off‐label use of PTH in human clinical trials suggests benefits for fracture healing fall short of the overwhelmingly positive results in animals. In particular, research on the effects of PTH in maxillofacial fracture healing is far from satisfactory. Since findings from animal studies and case reports are encouraging, future studies to examine the dose, timing, and duration of PTH administration are necessary to delineate the optimal therapeutic potential of PTH for fracture healing.

## Disclosures

Both authors state that they have no conflicts of interest.
